# Reverse transcriptase-PCR differential display analysis of meningococcal transcripts during infection of human cells: Up-regulation of *priA *and its role in intracellular replication

**DOI:** 10.1186/1471-2180-8-131

**Published:** 2008-07-29

**Authors:** Adelfia Talà, Mario De Stefano, Cecilia Bucci, Pietro Alifano

**Affiliations:** 1 Dipartimento di Scienze e Tecnologie Biologiche ed Ambientali, Università degli Studi del Salento, Via Monteroni, 73100 Lecce, Italy; 2 Dipartimento di Scienze Ambientali, Seconda Università degli Studi di Napoli, Via A. Vivaldi 43, 81100, Caserta, Italy

## Abstract

**Background:**

*In vitro *studies with cell line infection models are beginning to disclose the strategies that *Neisseria meningitidis *uses to survive and multiply inside the environment of the infected host cell. The goal of this study was to identify novel virulence determinants that are involved in this process using an *in vitro *infection system.

**Results:**

By using reverse transcriptase-PCR differential display we have identified a set of meningococcal genes significantly up-regulated during residence of the bacteria in infected HeLa cells including genes involved in L-glutamate transport (*gltT *operon), citrate metabolism (*gltA*), disulfide bond formation (*dsbC*), two-partner secretion (*hrpA-hrpB*), capsulation (*lipA*), and DNA replication/repair (*priA*). The role of PriA, a protein that in *Escherichia coli *plays a central role in replication restart of collapsed or arrested DNA replication forks, has been investigated. *priA *inactivation resulted in a number of growth phenotypes that were fully complemented by supplying a functional copy of *priA*. The *priA*-defective mutant exhibited reduced viability during late logarithmic growth phase. This defect was more severe when it was incubated under oxygen-limiting conditions using nitrite as terminal electron acceptors in anaerobic respiration. When compared to wild type it was more sensitive to hydrogen peroxide and the nitric oxide generator sodium nitroprusside. The *priA*-defective strain was not affected in its ability to invade HeLa cells, but, noticeably, exhibited severely impaired intracellular replication and, at variance with wild type and complemented strains, it co-localized with lysosomal associated membrane protein 1.

**Conclusion:**

In conclusion, our study i.) demonstrates the efficacy of the experimental strategy that we describe for discovering novel virulence determinants of *N. meningitidis *and ii.) provides evidence for a role of *priA *in preventing both oxidative and nitrosative injury, and in intracellular meningococcal replication.

## Background

*Neisseria meningitidis *(meningococcus) is a transitory colonizer of the human nasopharynx that sporadically provokes life-threatening disease. This microorganism has to interact with cellular barriers for its life cycle [1]. After crossing the nasopharyngeal mucosa, meningococci occasionally spread into the blood stream before moving across the blood-brain barrier causing fatal sepsis and meningitis in otherwise healthy individuals [2-5].

The early stages of an infection with *N. meningitidis *are governed by specific interactions between the pathogen and the epithelial tissues and are quite well known. Indeed, this microorganism has evolved a diverse array of surface structures subjected to phase- and antigenic-variation, which promote adherence and entry into human cells, although it is still unclear whether cell invasion is important in human infections. Initial adherence of encapsulated bacteria requires type IV pili, fine hair-like structures protruding from the bacteria surface [3]. Then, interaction with host cells is achieved by several meningococcal surface adhesins (Opa and Opc) that have been extensively studied [1,6]. In contrast, later stages of infection, including the intracellular location of the meningococci and their strategies for intracellular survival, are only poorly understood [1].

*In vitro *studies with cell line infection models are beginning to disclose the essential role of the metabolic adaptation of meningococci to the intracellular environment of the infected cell. The adaptive response includes the stimulation of the capsular biosynthetic genes leading to increased resistance to cationic antimicrobial peptides (CAMPs), important components of the host innate defense system against microbial infections [7], and the activation of the GdhR regulon whose members are involved in metabolism of the available host carbon sources [8,9].

In this work by using reverse transcriptase-PCR differential display (RT-PCR-DD) we have identified a set of meningococcal genes up-regulated during residence of the bacteria in the intracellular host environment including genes involved in L-glutamate transport (*gltT *operon), citrate metabolism (*gltA*), disulfide bond formation (*dsbC*), two-partner secretion (*hrpA-hrpB*), capsulation (*lipA*), and DNA replication/repair (*priA*). PriA is a single-stranded DNA-dependent ATPase, and a 3' to 5' DNA translocase/helicase that was discovered originally because of its requirement *in vitro *for the conversion of bacteriophage phiX174 viral DNA to the duplex replicative form [10,11]. In *Escherichia coli *this protein, at the crossroads of DNA replication and recombination, plays a central role in origin-independent, replication restart of collapsed or arrested DNA replication forks and is also involved in DNA recombination [12-14]. These activities rely on the ability of PriA to load replication forks at a D loop, an intermediate that forms during homologous recombination, double-strand break-repair, and stable DNA replication. We investigated the role of *priA *in the meningococcal infectious cycle using a human cell line infection model.

## Results

### RNA differential display analysis of meningococcal gene expression in the intracellular host environment

In an attempt to identify meningococcal genes selectively up- or down-regulated in the intracellular environment of infected host cells we used a previously published RT-PCR-DD strategy that relies on the presence of highly and moderately repetitive transcribed DNA sequences in the meningococcal genome [8]. Oligonucleotides designed on the basis of the DUS (DUS-IN and DUS-OUT) or the 26L, 27L *nemis *sequences (26L-IN, 26L-OUT, 27L-IN, 27L-OUT) were used as primers in reverse-transcriptase (RT) assays to prepare cDNAs from intracellular meningococci (strain B1940) recovered from saponin-lysed HeLa cells after 7 h of infection or control bacteria grown in DMEM without HeLa cells as previously described [7]. After 7 h of infection bacterial recovery from infected cells reached the highest values [7]. We decided to use HeLa cells because studies with these cells have contributed to our understanding of the molecular mechanisms underlying the infectious cycle of *N. meningitidis *[15-20]. The analysis of the meningococcal transcriptome during the early stages of an infection has been previously performed using HeLa cells [21].

**Figure 1:**
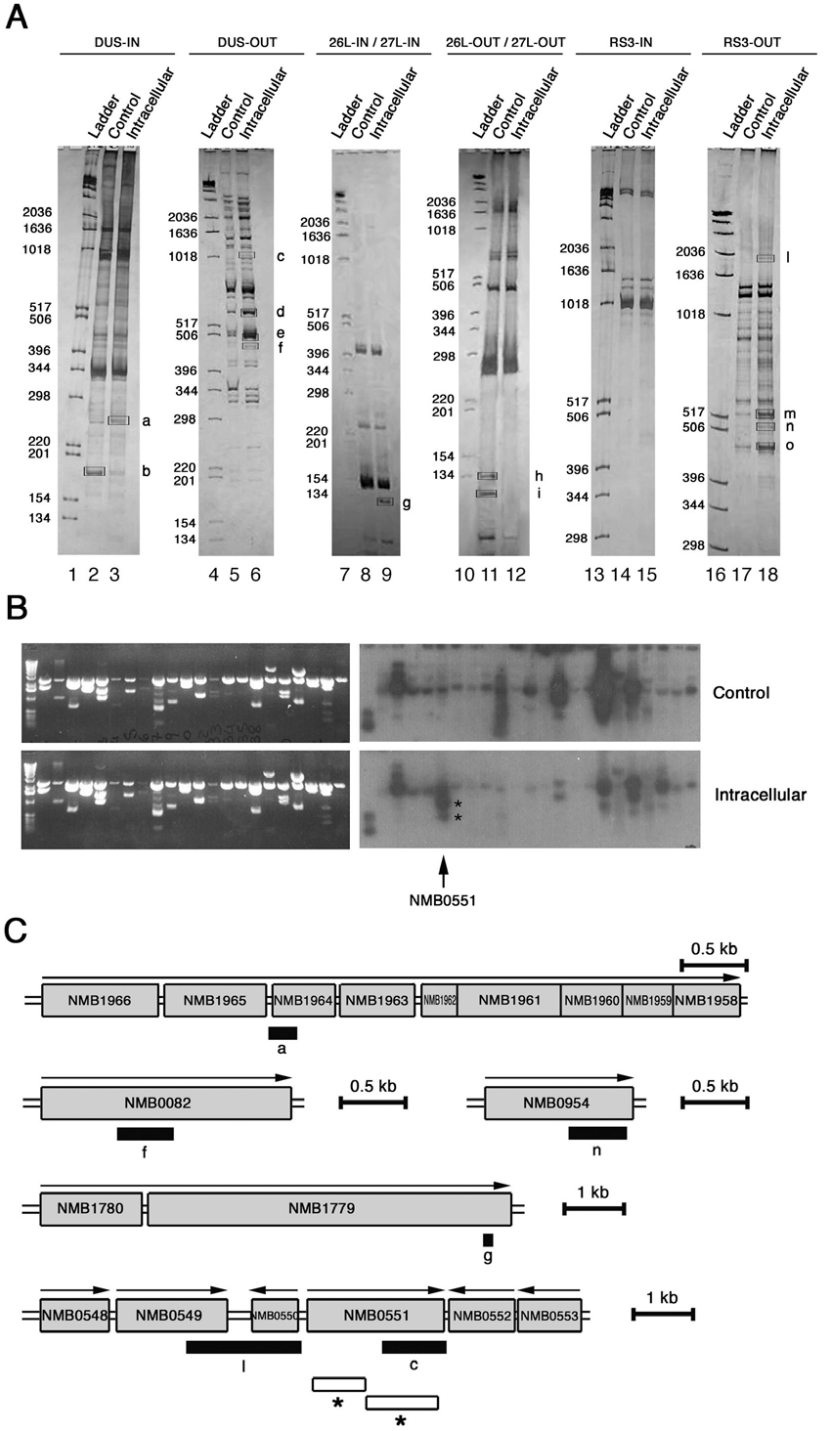
**RT-PCR-DD and limited transcriptional analysis of meningococcal transcripts up- and down-regulated during the intracellular phase**. (A) RT-PCR-DD analysis. Oligonucleotides DUS-IN, DUS-OUT, RS3-IN, RS3-OUT or oligonucleotide mixtures 26L-IN/27L-IN, 26L-OUT/27L-OUT were used as primers in first-strand cDNA synthesis using total RNAs from intracellular meningococci (strain B1940) recovered from saponin-lysed HeLa cells after 7 h of infection (intracellular) or control bacteria grown for 7 h in cell culture medium without HeLa cells (control) as templates. Then second-strand cDNAs synthesis was carried out using the corresponding oligonucleotides and a mixture of random hexamers as primers, and the PCR products were analyzed by polyacrylamide gel electrophoresis. In lanes 1 and 6 molecular weight ladders, whose sizes are indicated on the left of each panel, were run in parallel. The arrows indicate bands corresponding to either up-regulated (**a**, **c**, **d**, **e**, **f**, **g**, **l**, **m**, **n**, **o**) or down-regulated genes (**b, h, i**) in the intracellular environment. Molecular weight DNA ladders were run in parallel. (B) Limited transcriptional analysis. A partial *Sau3*AI-restricted genomic library from the strain B1940 was constructed. Individual plasmid clones were digested with *Sau3*AI and a Southern blot analysis was performed using ^32^P-labeled cDNA probes derived from intracellular bacteria after 8 h of infection of HeLa cells or control bacteria grown for 8 h in cell culture medium. In the figure only a limited number of clones (22 clones out of 3000) on duplicate filters are shown. The arrow indicates the cloned NMB0551 ORF corresponding to *priA*, up-regulated by intracellular meningococci. Asterisks mark the 983 and 1123 bp-long *Sau*3AI fragments contained in the plasmid clone. (C) Genetic map of meningococcal genes up-regulated in the intracellular environment. The positions of the cDNAs (closed rectangles) corresponding to up-regulated genes that were identified by RT-PCR-DD (**a, c, g, f, l, n**) are indicated with respect to the available genetic map of MC58. Open rectangles with asterisks locate the 983 and 1123 bp-long *Sau*3AI fragments contained in the *priA *plasmid clone shown in panel B.

The cDNAs from intracellular meningococci were then amplified by PCR using the corresponding oligonucleotides and a mixture of random hexamers as primers, and the PCRs were analyzed by polyacrylamide gel electrophoresis. By this approach, several bands corresponding to either up-regulated (**a**, **c**, **d**, **e**, **f**, **g**, **l**, **m**, **n**, **o**) or down-regulated genes (**b, h, i**) were detected in the intracellular bacteria (Fig. 1A). The bands **a**, **c**, **f**, **g**, **l **and **n **were then excised from the gels, the corresponding cDNAs were cloned and subjected to nucleotide sequences analysis. Three clones were sequenced form each band. The bands **d**-, **e**-, **m**-, and **o**-associated cDNAs, when subjected to cloning, each gave rise to more than one clone, and therefore they were not further analyzed in this study. Bands corresponding to down-regulated genes were not examined.

The nucleotide sequence analysis demonstrated that the band **a**- and **f**-associated cDNA corresponded to genes that have been shown to be induced in the intracellular environment and have been also implicated in meningococcal pathogenesis (Fig. 1C). The band **a**-associated cDNA corresponded to NMB1964, a gene of the GdhR-regulated *gltT *operon coding for the L-glutamate ABC transporter that is critical for meningococcal adaptation in the low-sodium intracellular environment [8]. The band **f **corresponded to NMB0082 coding for LipA, a protein required for proper translocation and surface expression of the lipidated (α2→8)-linked polysialic acid capsule polymer [7,22]. The band **g**-associated cDNA sequence fell within NMB1780-NMB1779 (*hrpB-hrpA*) operon coding for a two-partner secretion (TPS) system that has been recently shown to contribute to adhesion of un-encapsulated bacteria to epithelial cells [23]. The bands **c**-, **l**- and **n**-associated cDNAs corresponded to genes whose role in meningococcal pathogenesis has never been reported so far, including NMB0551 (band **c **sequence) encoding PriA, the linked NMB0549 and NMB0550 (band **l **sequence) coding, respectively, for a thiol-disulfide oxidoreductase (DsbC) and the ATP-binding protein of an unknown ABC-type transporter (YbjZ), and NMB0954 (band **n **sequence) encoding GltA, the putative meningococcal citrate synthase (Fig. 1C). Fig. 1C also shows the genes surrounding *priA *(NMB0551). Upstream from NMB0549 (*ybjZ*) a gene coding for an AcrA/AcrE family protein (NMB0548) is located; downstream to *priA *and in the opposite direction a gene coding for a protein of unknown function (NMB0552) and that for a putative transposase (NMB0553) map.

### The role of priA in meningococcal pathogenesis

The role of PriA in meningococcal pathogenesis was explored in more detail using the HeLa infection model. It is noteworthy that over-expression of *priA *was also observed in an independent screening by limited transcriptional analysis (Fig. 1B) that was useful to demonstrate up-regulation of *lipA *in a previous work [7]. This screening was performed using a partial *Sau3*AI-restricted genomic library from B1940. The library was screened by Southern blot using cDNA probes derived from intracellular bacteria after 8 h of infection of HeLa cells or control bacteria grown for 8 h in cell culture medium. The screening led to isolation of a plasmid harboring two *Sau3*AI fragments, 983 and 1123 bp-long (marked by asterisks in Fig. 1B and 1C), spanning almost the entire *priA *gene.

**Figure 2:**
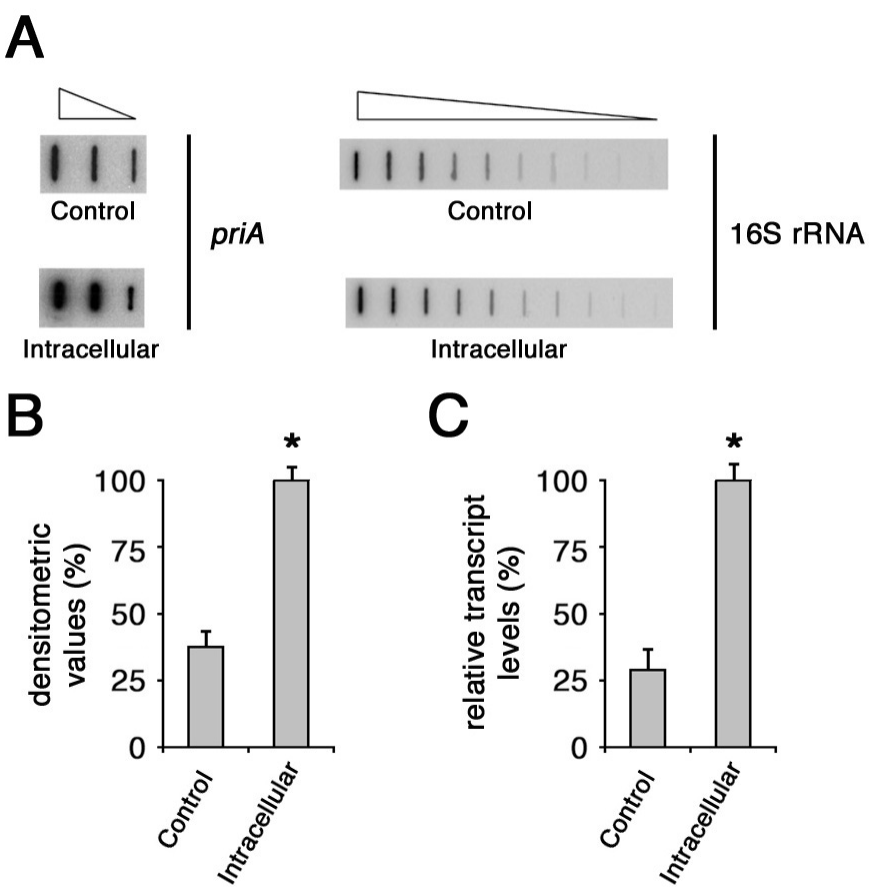
**Semi-quantitative analysis of *priA*-specific transcripts in intracellular meningococci**. (A) RT-PCR slot blot analysis of the *priA*-specific transcripts. Total RNAs were extracted from meningococci (strain B1940) after 7 h-infection of HeLa cells (intracellular), or control bacteria grown for 7 h in culture medium (control) and were used to generate gene-specific ^32^P-labeled cDNA probes as detailed in the Methods section. The ^32^P-labeled cDNA probes were hybridized to different amounts (4, 20 and 100 ng) of denatured NMB0551 (*priA*)-specific DNA fragments. For the 16S rRNA gene-specific fragment, two-fold serial dilutions (from 0.05 to 50 ng) were used. (B) Densitometry analysis of the RT-PCR slot blot with 20 ng of *priA*-specific DNA fragments. The relative transcript levels of *priA *in intracellular meningococci are arbitrarily assumed equal to 100%. Values represent means from five independent experiments, each with triplicate samples, using RNA preparations from distinct infection assays. Bars indicate standard deviations. (C) Semi-quantitative analysis of the *priA*-specific transcripts by RT real-time PCR experiment. The RNAs were extracted from intracellular or control meningococci as described above. Results were normalized to 16S rRNA levels. Transcript levels of *priA *in intracellular meningococci were arbitrarily given a value of 100%. Data are shown as mean ± standard deviation from five independent experiments, each with triplicate samples, using RNA preparations from distinct infection assays. The Student's T-test was used for statistical analysis. Statistically significant differences between values from intracellular and control bacteria (asterisks) are declared at a p value < 0.05.

Nevertheless, in order to confirm the results of the RT-PCR-DD analysis, slot blot and RT real-time PCR experiments were performed (Fig. 2). Semi-quantitative analysis by slot blot experiments demonstrated that the *priA*-specific RNA was about three-fold more abundant in meningococci recovered from saponin-lysed HeLa cells than in control bacteria grown in DMEM without HeLa cells (Fig. 2A–B). This result was confirmed by RT real-time PCR demonstrating an about four-fold increase in the amount of *priA *transcripts in intracellular meningococci (Fig. 2C). It should be noted that RT-PCR-DD, limited transcriptional analysis and RT real-time PCR were performed on RNAs from different sets of infection experiments thus enhancing the argument for a role of this gene in the intracellular environment.

**Figure 3:**
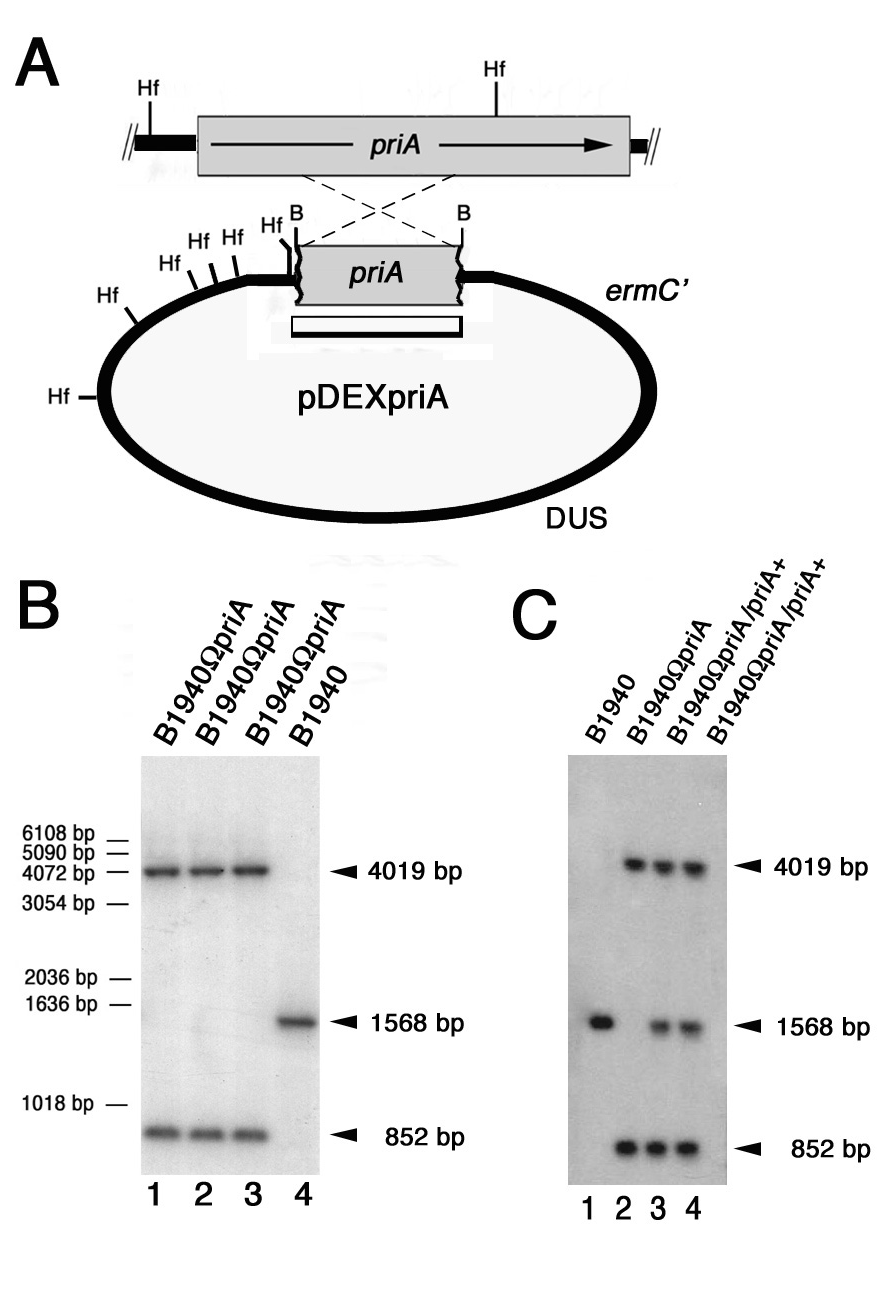
**Knockout of NMB0551 (*priA*) and complementation**. (A) Experimental design for *priA *disruption by single cross over. The genetic and physical map of the *priA *gene is depicted above the map of the pDEXpriA region involved in single cross-over and the probe (open rectangle) used in the Southern blot experiments shown in panels B and C. *ermC'*, erythromycin-resistance gene; DUS, DNA uptake sequence; B, *Bam*HI restriction sites; Hf, *Hin*fI restriction sites. (B) Southern blot analysis demonstrating the inactivation of *priA*. Chromosomal DNAs were extracted from the parental strain B1940 (lane 4) and from three recombinant strains (B1940ΩpriA) transformed with pDEXpriA (lanes 1–3). The *Hin*fI-restricted chromosomal DNAs were analyzed by Southern blot using the ^32^P labeled *priA*-specific DNA fragments cloned in pDEXpriA as probes. The arrows on the right indicate *priA*-specific fragments whose sizes were deduced on the basis of the relative migration of DNA ladders (bars on the left). (C) Southern blot experiment demonstrating complementation of B1940ΩpriA. B1940ΩpriA was transformed with pACpriA harboring a functional copy of *priA*, and chromosomal DNAs were extracted from two recombinant strains (B1940ΩpriA/priA+) (lanes 3 and 4). The DNAs from these two strains, from B1940 (lane 1) and from B1940ΩpriA (lane 2) were digested with *Hin*fI and analyzed as in panel B.

To gain functional information about the role of *priA *in *N. meningitidis*, we decided to inactivate it insertionally by transformation with pDEXpriA (Fig. 3A and Materials and Methods). Southern blot analysis confirmed the insertion of this suicide plasmid into the *priA *coding region by a single cross-over event (Fig. 3B). As a result of the recombination event, two *Hin*fI DNA fragments of the expected sizes (4019 bp and 852 bp) were detected by *priA*-specific probe in the transformed strains B1940ΩpriA (Fig. 3B, lanes 1–3) in place of the 1568 bp *Hin*fI fragment of the parental strain B1940 (Fig. 3B, lane 4). In order to be sure that *priA *inactivation was causal to all of the phenotypes observed below, a functional copy of *priA *was introduced into *leuS*-*Ψdam *region of B1940ΩpriA by using the integrative plasmid pACpriA. Successful gene delivery was demonstrated by Southern blot analysis (Fig. 3C). In two transformants (B1940ΩpriA/priA+) the *priA*-specific probe detected the presence of both the 1568 bp *Hin*fI fragment (typical of the wild type *priA*) and the 4019 bp and 852 bp *Hin*fI fragments (typical of the inactivated *priA*) (Fig. 3C, lanes 3 and 4).

**Figure 4:**
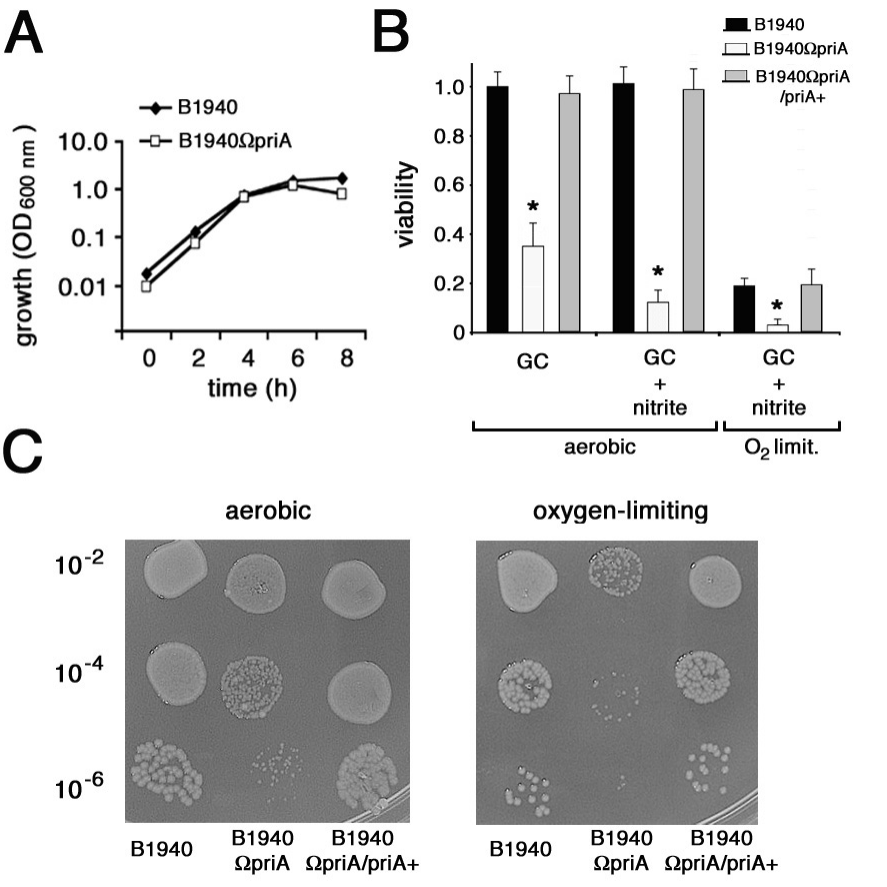
**Effects of *priA *inactivation on meningococcal growth**. (A) Growth curves of B1940 and B1940ΩpriA in liquid GC medium. (B and C) Viability of meningococcal cells as determined by CFU method. Serial dilutions (10^-2 ^to 10^-6^) of B1940, B1940ΩpriA and B1940ΩpriA/priA+ cultures in liquid GC medium were plated on either GC agar or GC agar containing 5 mM sodium nitrite and incubated aerobically in the presence of 5% CO_2_; parallel dilutions were plated on GC agar containing 5 mM sodium nitrite and incubated under oxygen-limiting conditions. CFU were determined after 24 h. Viability of B1940 grown aerobically is arbitrarily assumed equal to 1.0. Data are shown as mean ± standard deviation from five independent experiments, each with triplicate samples. The Student's T-test was used for statistical analysis. Statistically significant differences between values from B1940 (or B1940ΩpriA/priA+) and B1940ΩpriA (asterisks) are declared at a p value < 0.05. Note in C the small colony size and the reduced colony number of the *priA*-defective mutant compared to the wild type and complemented strains.

Phenotypic analysis demonstrated that *priA *inactivation resulted in a growth defect. Indeed, under aerobic conditions, B1940ΩpriA strain failed to grow in diluted inocula (= or <10^6 ^CFU ml^-1^), but when it was inoculated at higher densities (= or >10^7 ^CFU ml^-1^) it grew at rates similar to those of the parental strain (Fig. 4A). However, in high-density inocula viability of B1940ΩpriA as determined by CFU was apparently reduced when compared to that of B1940. In the experiment shown in Fig. 4C, left panel, B1940, B1940ΩpriA and B1940ΩpriA/priA+ were grown aerobically in liquid GC medium to late logarithmic phase (1.0 OD_600 nm_). Then, serial dilutions of the cultures were plated on GC agar, incubated aerobically in the presence of 5% CO_2_, and the number of CFU determined after 24 h. Results demonstrated small colony size (Fig. 4C) and reduced colony number (to about 34%) of the *priA*-defective strain compared to the parental one (Fig. 4B). This phenotype was much more severe when GC agar plates containing nitrite were incubated under either aerobic (not shown) or oxygen-limiting conditions (Fig. 4C, right panel). Under both these conditions the colony number of B1940ΩpriA was reduced to about 15% when compared to that of B1940 under the same conditions (Fig. 4B). Statistical analysis with the Student's T-test confirmed that the growth defects in the presence of nitrite or in oxygen limited conditions in the presence of nitrite were more severe than under aerobic conditions without nitrite (p value < 0.05). The growth defect of B1940ΩpriA was not observed during middle logarithmic phase (data not shown), and could be completely restored by complementation with pACpriA in B1940ΩpriA/priA+ (Fig. 4B and 4C).

**Figure 5:**
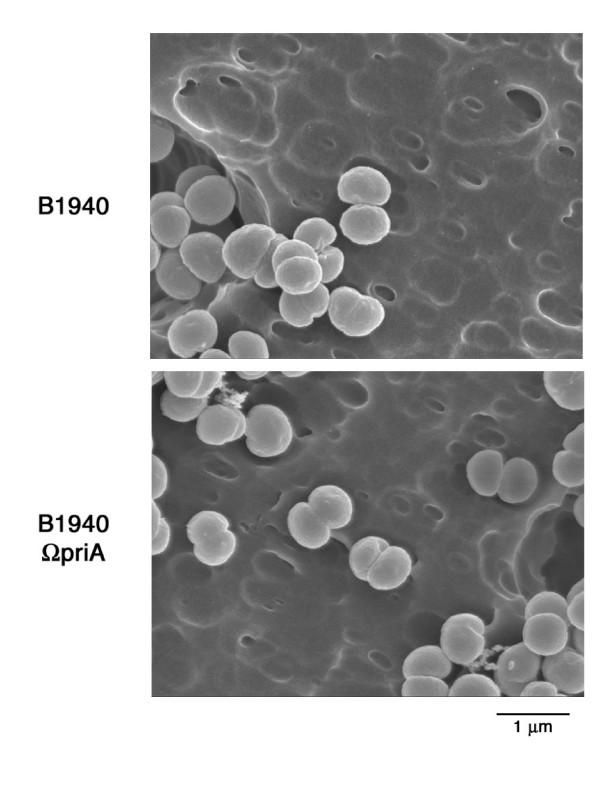
**Morphology of B1940 and B1940ΩpriA meningococci**. Meningococcal strains B1940 and B1940ΩpriA were grown to late (1.0 OD_600 nm_) logarithmic phase and observed by scanning electron microscopy. Note that *priA*-inactivation does not affect the typical diplococcal, coffee bean-shaped morphology of meningococci.

We next investigated the mechanism underlying the growth defect of B1940ΩpriA. As there is evidence that in *E. coli priA *inactivation results in defective cell division that generates long filaments [24], we used scanning electron microscopy (SEM) to analyze morphology B1940ΩpriA. Indeed, the growth defect of this strain as determined by CFU method might have been caused by a cell division defect that in meningococci, which divide in alternating planes generates clumps. However, SEM analysis did not reveal any difference between the wild type and the *priA*-defective strain grown to late logarithmic phase (1.0 OD_600 nm_) (Fig. 5).

**Figure 6:**
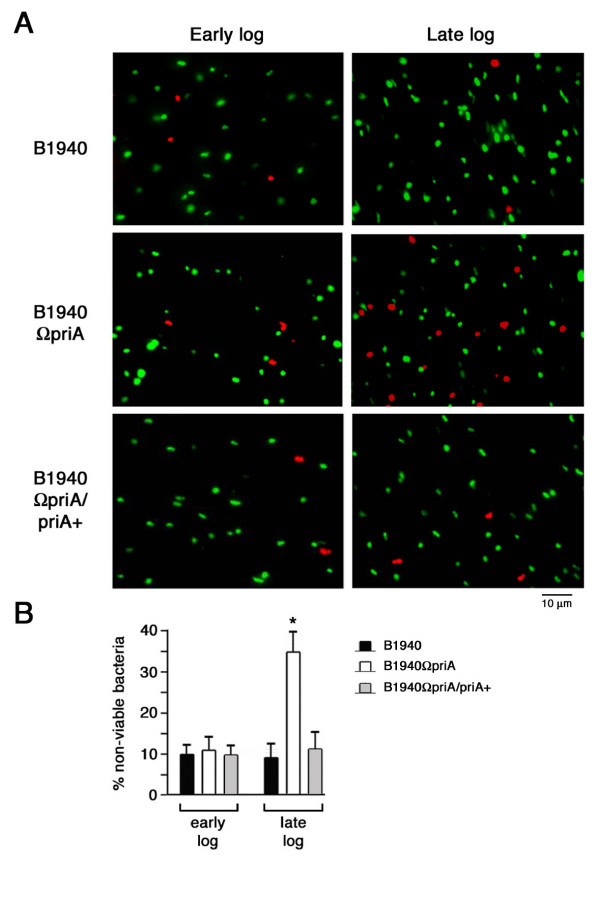
**Effects of *priA *inactivation on meningococcal viability**. Meningococcal strains B1940, B1940ΩpriA and B1940ΩpriA/priA+ were grown to either middle (0.5 OD_600 nm_) or late (1.0 OD_600 nm_) logarithmic phase and then viability was determined by using the Live/Dead Bac*Light *bacterial viability kit (Molecular Probe). (A) Representative images of Live/Dead-stained meningococci: live bacteria fluoresce green, while dead bacteria fluoresce red. (B) Percentages of non-viable meningococci were equal to ratios between bacteria staining positive with propidium iodide (red) and the total stained (red + green) bacteria. Data are shown as mean ± standard deviation of three experiments. Statistically significant difference between values from B1940 (or B1940ΩpriA/priA+) and B1940ΩpriA (asterisk) is declared at a p value < 0.05.

To confirm the hypothesis that the growth impairment of B1940ΩpriA might be due to reduced viability, the Live/Dead staining assay was carried out. This assay employs two fluorescent nucleic acid stains, SYTO9 and propidium iodide, which differ in their ability to penetrate healthy bacterial cells. When used alone, the green-fluorescent SYTO 9 stain labels both live and dead bacteria. In contrast, the red-fluorescent propidium iodide stain penetrates only bacteria with damaged membranes, reducing SYTO 9 fluorescence when both dyes are present. Thus, live bacteria fluoresce green, while dead bacteria fluoresce red (Fig. 6A). Using this system we did not observe any significant changes in viability between B1940 and B1940ΩpriA when stain was carried out with bacteria grown in GC medium to middle logarithmic phase (0.5 OD_600 nm_). Indeed, the percentage of nonviable cells was approximately 10% for both strains. In contrast, B1940ΩpriA exhibited marked loss of viability during late logarithmic phase (1.0 OD_600 nm_) when the percentage of nonviable cells approximated 35% (Fig. 6B). The defect in viability was completely restored by complementation (Fig. 6A and 6B). The Live/Dead staining assay also confirmed the absence of any cell division defect in B1940ΩpriA (Fig. 6A).

**Figure 7:**
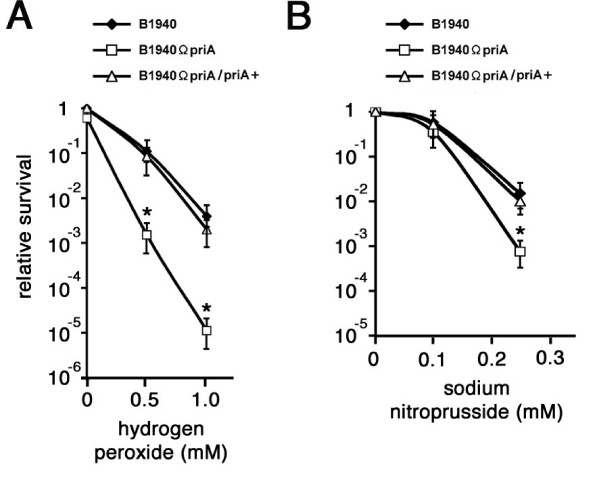
**Effects of *priA *inactivation on sensitivity to oxidative injury**. (A-B) B1940, B1940ΩpriA and B1940ΩpriA/priA+ were grown to middle logarithmic phase (0.5 OD_600 nm _corresponding to about 1 × 10^9 ^CFU for both strains) and then exposed for the indicated times to hydrogen peroxide (A) or nitric oxide generator sodium nitroprusside (B) in GC liquid medium with shaking and then viability was determined by recovery of viable bacteria. Data are shown as mean ± standard deviation from three independent experiments, each with triplicate samples. Statistically significant differences between values from B1940 and B1940ΩpriA (asterisks) are declared at a p value < 0.05.

Assessment of viability after exposure to hydrogen peroxide (Fig. 7A) or the nitric oxide generator sodium nitroprusside (Fig. 7B) also demonstrated that B1940ΩpriA was much more sensitive to oxidative and nitrosative injuries when compared to the parental strain. The phenotype could be complemented back to wild type levels in B1940ΩpriA/priA+.

**Figure 8:**
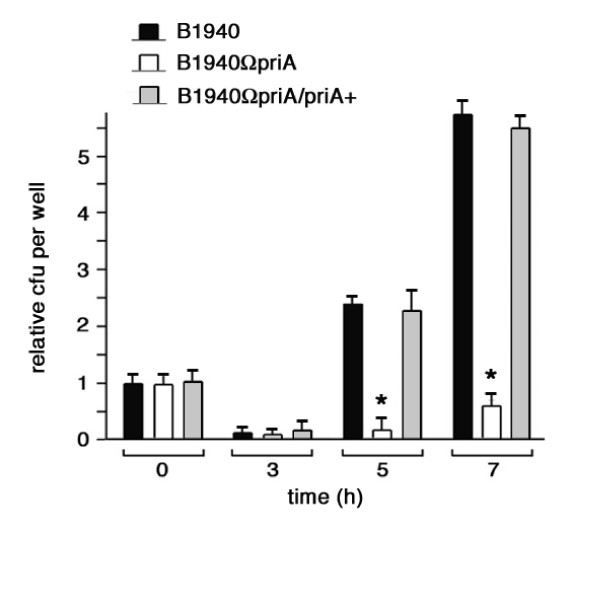
**Effects of *priA *inactivation on survival/growth of meningococcal strains in HeLa cells**. HeLa cells (10^5^/well) were infected with B1940, B1940ΩpriA or B1940ΩpriA/priA+ at a MOI of 50, treated with gentamicin and re-incubated in DMEM for the indicated times. Bacteria were centrifuged onto cells to start infection. After saponin lysis, CFU from intracellular bacteria were scored. Values are relative to the number of intracellular B1940 at time 0 (10^4 ^bacteria per 2 × 10^5 ^cells per ml) and are means of at least ten independent experiments made in triplicate with standard errors.

B1940, B1940ΩpriA and B1940ΩpriA/priA+ were then used in cell invasion and intracellular persistence assays. In these experiments, high density-inocula of meningococci were used and infections were initiated by using comparable numbers of viable bacteria for both strains as determined by both CFU method and Live/Dead staining. After allowing meningococci to invade HeLa cells for 1 h, intracellular viability was assessed immediately after gentamicin (time 0 h) treatment and 3, 5 and 7 h post-gentamicin treatment (Fig . 8). Results demonstrated the absence of statistically relevant differences in the ability to invade the HeLa cells by the three strains (Fig. 8, time 0 h). At 3 h post-gentamicin treatment the number of recoverable wild type, mutant and complemented bacteria dropped about ten-fold. Normally, at this time, a decrease occurs in intracellular viable bacteria, which is also characteristic of gonococcal invasion [7,8,25,26]. Between 3 and 7 h B1940 and B1940ΩpriA/priA+ underwent fast duplication and the number of viable bacteria increased more than 50-fold. In particular, intracellular duplication was extremely fast during the first 2 h after gentamicin treatment (time 3–5 h) with an apparent generation time of about 24 min and slower during the last 2 h (time 5–7) consistent with previous findings [7,8]. In contrast, between 3 and 7 h the number of viable B1940ΩpriA bacteria increased less than 10-fold with an apparent generation time of 96 min during the first 2 h after gentamicin treatment (time 3–5 h). It should be noted that intracellular generation times are only apparent, as they reflect the sum of intracellular growth, intracellular death and, eventually, bacterial egression from HeLa cells and re-invasion. Therefore different processes may be affected in this mutant.

**Figure 9:**
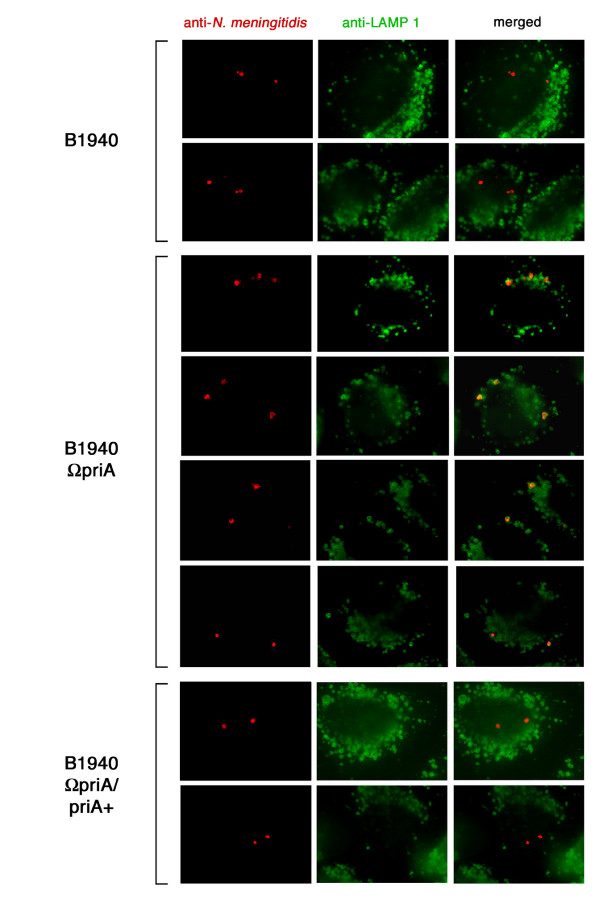
**Immunofluorescence analysis of HeLa cells during infection**. HeLa cells were infected with B1940, B1940ΩpriA or B1940ΩpriA/priA+ as indicated. Gentamicin selection was used to eliminate extracellular bacteria. Images were taken 7 h after infection. Antibodies against *N. meningitidis *were used before permeabilization with saponin in combination with a secondary antibody conjugated with Cy5 and, after permeabilization, with a secondary TRITC-conjugated antibody. Intracellular bacteria were therefore red while extracellular bacteria were expected to be purple (the combination of TRITC and Cy5 signal). In the images all bacteria were intracellular. To detect cellular markers we used anti-LAMP1 and a secondary antibody conjugated with FITC.

To further investigate the origin of the growth/survival defect of B1940ΩpriA inside HeLa cells we used immunofluorescence microscopy. Intriguingly, we noted that number of intracellular *priA*-defective bacteria 7 h after infection was about the same as those of wild type and complemented bacteria (Fig. 9 and data not shown). This seemed to be in contrast with the data obtained with the CFU method showing a marked decrease in the number of intracellular *priA*-defective bacteria 7 h after infection (Fig. 8). However, in contrast to CFU method, immunofluorescence analysis detects even dead bacteria, and this means that most *priA*-defective meningococci inside HeLa cells were actually not viable. The evidence that, at variance with wild type and complemented bacteria, most *priA *mutant bacteria co-localized with the lysosomal associated membrane protein 1 (LAMP1) strongly supported this hypothesis. This finding is noteworthy because live meningococci have been shown to use IgA protease to cleave LAMP1 protein [27], accounting for the lack of co-localization of wild type and complemented bacteria with this marker. On the basis of this evidence we concluded that the growth phenotype of the *priA*-defective mutant inside HeLa cells was mostly due to reduced viability than to reduced growth rate.

## Discussion

The goal of our study was to assess the reliability of RT-PCR-DD as a tool to identify genes with increased or decreased expression during meningococcal growth in infected cells. Although this analysis was clearly not exhaustive as the repeat sequences that we used to prime the RT-PCR-DD reactions were not randomly distributed in the chromosome, and not found in every gene [28], nevertheless it enabled us to identify three up-regulated genes/operons (*gltT*, *lipA, hrpA-hrpB*) with known roles in virulence (thus validating this approach) and three novel potential meningococcal virulence factors (Fig. 1). One of these, *priA*, was selected for extensive further validation (Fig. 2) and characterization.

In infected cells meningococci have to adapt their metabolism to the available host carbon and nitrogen sources and circumvent or subvert a number of host defense mechanisms including lysosomal killing, antimicrobial peptides and oxidative injury. Up-regulation of the *gltT *operon, coding for an L-glutamate ABC type transporter that is critical for meningococcal adaptation in the low-sodium intracellular environment [8], and of *gltA*, coding for the citrate synthase, catalyzing the first committed step of the citric acid cycle is part of a metabolic strategy to survive and grow in the intracellular environment. Indeed, in the intracellular milieu there is little available glucose, as glucose entering cells is rapidly phosphorylated to glucose-6-phosphate [29], a substrate that, as well as the other glycolytic intermediates, cannot be assimilated by meningococci. The best intracellular carbon sources are pyruvate and lactate or/and several amino acids such as L-glutamate that stimulate the citric acid cycle [26,30].

Stimulation of L-glutamate uptake and respiratory metabolism is also useful to prevent oxidative injury, a major source of DNA damage, as this amino acid is the precursor of glutathione [29]. In this context the up-regulation of thiol-disulfide oxidoreductase (DsbC)- and PriA-encoding genes (Figs. 1 and 2), and the impaired ability of the *priA*-defective mutant to replicate in infected HeLa cells (Figs. 8 and 9) may be relevant. PriA plays a central role in origin-independent replication restart of collapsed or arrested DNA replication forks [12-14]. There is evidence that, even under normal aerobic conditions, most, if not all, replication forks do not proceed from the origin of replication to the terminus without hitting a roadblock, in the form of a damaged DNA template, a nick in the template, a "frozen" protein-DNA complex, or local positive superhelicity, which have the potential to arrest or collapse the fork. Inability to reassemble the replisome and resume replication in a timely manner after repair of DNA damage can lead to chromosomal rearrangements and consequently, a loss in viability [31].

Oxidative injury is a major cause of DNA replication fork arrest [32]. This accounts for the growth defect mostly due to reduced viability of the *priA*-defective meningococcal strain (Figs. 4 and 6), which was much more sensitive to oxidative and nitrosative injuries when compared to the parental strain (Fig. 7). Reduced viability was also observed in *E. coli priA*-defective mutants [24,33]. A growth defect was also shown in the *Neisseria gonorrhoeae priA*-defective mutant [34]. This strain was also defective in DNA repair and DNA transformation compared to the isogenic parental strain. However, based on staining with Live/Dead Bac*Light *kit the authors concluded that the growth defect could not be attributable to reduced viability. The different biology of the meningococcal and gonococcal infection may account for the different phenotype of the meningococcal and gonococcal *priA*-defective mutants. Indeed, substantial differences in oxidative defenses between meningococci and gonococci have been reported. When compared to meningococci, gonococci that are usually associated with inflamed urogenital tissues and activated polymorphonuclear leukocytes, have much higher catalase and cytochrome c peroxidase activities in addition to a specific manganese (II) uptake system involved in resistance to superoxide radicals [35].

The growth defect of the *priA*-defective meningococcal strain was more severe in the presence of nitrite under either aerobic or oxygen-limiting growth conditions (Fig. 4). Under these conditions meningococci use nitrite as a terminal electron acceptor in anaerobic respiration. Nitrite reduction via nitric oxide to nitrous oxide is the partial denitrification pathway for this mode of growth. In pathogenic *Neisseriae *the pathway involves both a copper-containing nitrite reductase (AniA) and a single subunit nitric oxide reductase (NorB) [36,37]. Nitrite reduction leads to the production of nitric oxide, a toxic molecule that exhibits synergistic activity in combination with reactive oxygen species [38] and that in *Salmonella *induces DNA replication arrest [39]. The inability to restart nitric oxide-promoted arrested replication forks may account for reduced viability of the *priA*-defective mutant under oxygen-limiting conditions.

The relevance of nitrite reduction and defence mechanisms against nitric oxide injury in neisserial pathogenesis is the subject of investigation. Nitrite is present in the diet and is naturally formed in human blood and tissues due to the oxygenation of nitric oxide that is produced by various cell types as a signaling molecule and as a weapon against pathogens and tumor cells. It has been proposed that in microaerobic environments including the human nasopharynx and in the nitric oxide-enriched intracellular environment, meningococcal partial denitrification contributes with oxygen respiration as a route for electron transfer in respiration [37,40]. Interestingly, inside HeLa cells at variance with wild type and complemented bacteria, *priA*-defective meningococci co-localized with LAMP1 (Fig. 9) that marks highly oxidative and nitrosative cell compartments [41]. Based on this evidence, it is reasonable to assume that failure of the *priA*-defective mutant to replicate efficiently in HeLa cells may reflect the reduced ability to restart arrested replication forks.

## Conclusion

In conclusion, in this study we, on one hand, demonstrate the efficacy of the experimental strategy that we describe, i.e. the analysis of the meningococcal gene expression, for disclosing the mechanisms that *N. meningitidis *uses to survive and multiply inside the environment of the human cell. On the other hand, we provide evidence that *priA*, whose expression increases in the intracellular environment, plays a key role in preventing both oxidative and nitrosative injuries, and in intracellular meningococcal replication.

## Methods

### Bacterial strains and growth conditions

*N. meningitidis *serogroup B strain B1940 was used in this study. The origin and genotype of this strain have been previously reported [42]. Meningococci were cultured either aerobically in GC broth or agar with 1% Polyvitox, at 37°C in a 5% CO_2 _incubator or under oxygen-limiting conditions in GC agar containing 5 mM sodium nitrite with 1% Polyvitox, at 37°C in an anaerobic jar with an anaerobic atmosphere generator (GENbox anaer, Biomerieux) and an indicator (Anaer indicator, Biomerieux).

*E. coli *strain DH5α [F^-^Φ80d *lacZ*ΔM15 *endA1 recA1 hsdR17 supE44 thi*-1 λ^- ^*gyrA96 *Δ(*lacZYA-argF*) *U169*] was used in cloning procedures. This strain was grown in Luria Bertani (LB) medium. To allow plasmid selection, the LB medium was supplemented with ampicillin (50 μg ml^-1^).

### Cell culture and infection

For standard invasion and intracellular viability assays, HeLa cells (ATCC Number CCL-2) were used. Several experiments were also repeated with HEp2 (ATCC Number CCL-23) and Chang conjunctiva cells (ATTC Number CCL-20.2). It should be noted, however, that, as a result of a well known contamination event, HEp2 (thought to be derived from an epidermoid carcinoma of the larinx) and Chang conjunctiva (thought to be derived from normal conjunctiva) cells present HeLa markers and have been established via HeLa cells contamination, as stated by ATCC . These cells were grown at 37°C in a 5% CO_2 _incubator in DMEM supplemented with inactivated 10% fetal bovine serum (FBS), 2 mM L-glutamine, penicillin (50 U ml^-1^) and streptomycin (50 μg ml^-1^).

*N. meningitidis *invasion assays were performed as previously described [8,7]. In brief, HeLa (or HEp2 or Chang conjunctiva) cells were infected at a multiplicity of infection (MOI) of 50 for 1 h. To start the infection bacteria were centrifuged (60 × g) onto cells. Cells were washed twice with phosphate-buffered saline (PBS) to eliminate the majority of extracellular bacteria and exposed to gentamicin to kill remaining extracellular bacteria. Cells were then washed extensively with PBS to remove gentamicin and dead extracellular bacteria, and then lysed with saponin. Gentamicin treatment was performed at 100 μg ml^-1^, a concentration 10-fold above the minimal inhibitory concentration (MIC) for 30 min. When required, cells were re-incubated in a fresh culture medium for various time intervals after gentamicin treatment. For quantification, bacteria released by saponin from HeLa cells (intracellular) were plated and colony-forming units (CFU) were counted the day after.

### DNA procedures and genetic manipulation

High-molecular-weight genomic DNA from *N. meningitidis *strains was prepared as described previously [43]. Oligonucleotides used in this study as primers in PCRs are listed in Table 1. Oligonucleotide synthesis was performed as a service by MWG-Biotech AG Oligo Production. The amplification reactions generally consisted of 35 cycles including 45 s of denaturation at 94°C, 45 s of annealing at 65°C, and 45 s of extension at 72°C. They were carried out in a Perkin-Elmer Cetus DNA Thermal Cycler 2400. DNA from strain B1940 was used as a template. Southern blot hybridizations were carried out according to standard protocols [44]. ^32^P labeling of the DNA fragments was performed by random priming using the Klenow fragment of the *E. coli *DNA polymerase I and [α-^32^P]dATP and [α-^32^P]dGTP (3,000 Ci mmol^-1^) [44]. DNA sequencing was performed as a service by the MWG Biotech Custom Sequencing Service. Processing of the DNA sequences was performed with the software GeneJockey Sequence Processor (published and distributed by Biosoft). Deduced amino acid sequence similarity searching was performed with the BLAST program using the Conserved Domain Database available at the NCBI.

**Table 1:** Oligonucleotides used in this study

**Name**	**Sequence**^a^
NMB0551-1	5'-GATTGGGCGGATCCGGGTTGGATTGAAACAACGGAAGCG-3'
NMB0551-2	5'-CGTTGGTGCAGGATCCTTTTGGCGGAGCAGTTCGGGCAGCC-3'
NMB0551-f	5'-GCAGGTGTTGTTTCTGTTGCCC-3'
NMB0551-r	5'-CAAATAATCCTGCGTGCGCTTG-3'
NMB0551-3	5'-GAATAGGTCTAGACCAAACGCGCTTTCAAAGAGGCGG-3'
NMB0551-4	5'-GGGCGAAAGCTTTTACAGACGATCCGGAATAAAAATG-3'
DUS-IN	5'-GCCGTCTGAA-3'
DUS-OUT	5'-TTCAGACGGC-3'
26L-IN	5'-GTGGATTAACAAAAATCAGGAC-3'
26L-OUT	5'-GTCCTGATTTTTGTTAATCCAC-3'
27L-IN	5'-GTGGATTAAATTTAAATCAGGAC-3'
27L-OUT	5'-GTCCTGATTTAAATTTAATCCAC-3'
RS3-IN	5'-CATTCCCGCGCAGGCGGGAATC-3'
RS3-OUT	5'-GATTCCCGCCTGCGCGAATG-3'
16Suniv-1	5'-CAGCAGCCGCGGTAATAC-3'
16Suniv-2	5'-CCGTCAATTCCTTTGAGTTT-3'
16S-r	5'-CTACGCATTTCACTGCTACACG-3'

^a^Restriction sites *Xba*I and *Hin*dIII are underlined.

The *Neisseria-E. coli *shuttle vectors pDEX and pACNL1 have been previously described [9,45]. pACNL1 is a pACYC184 derivative harboring a 1480 bp *Sau*3AI fragment, spanning the meningococcal *leuS*-*Ψdam *region in the *Bam*HI site. To construct pDEXpriA, the DNA corresponding to the central segment of the ORF NMB0551 was amplified using the primers NMB0551-1 and NMB0551-2, and the *Bam*HI-restricted 851 bp PCR product was cloned into the *Bam*HI site of pDEX. NMB0551 (*priA*) was inactivated in B1940 by single cross-over event using pDEΔpriA originating the strain B1940ΩpriA. Transformations were performed by using 0.1 to 1 μg of plasmid DNA. Transformants were selected on GC agar medium supplemented with erythromycin (7 μg ml^-1^). Successful gene inactivation was demonstrated by Southern blot hybridization. NMB0551-specific probe was obtained by ^32^P labeling of *Bam*HI-restricted PCR fragment. The size of this fragment was 851 bp (primers NMB0551-1 and NMB0551-2). B1940ΩpriA was complemented by transformation with the integrative plasmid pACpriA. To construct this plasmid, the *priA *gene (with flanking regulatory elements) was amplified from strain B1940 using the primers NMB0551-3 and NMB0551-4 (Table 1). The *Xba*I/*Hin*dIII-restricted 2565 bp PCR product was cloned into the *Xba*I/*Hin*dIII sites of pACNL1. Transformants were selected on GC agar medium supplemented with chloramphenicol (5 μg ml^-1^).

### RNA procedures

Total RNAs were extracted from intracellular bacteria during the infection of HeLa cells or control bacteria grown in culture medium as described [8].

The procedure for limited transcriptional analysis has been previously detailed [9,8]. Briefly, a partial *Sau3*AI-restricted genomic library from the serogroup B strain B1940 was constructed. Individual clones were digested and a Southern blot analysis was performed using cDNA probes derived from intracellular bacteria after 8 h of infection of HeLa cells or control bacteria grown for 8 h in cell culture medium. This procedure detects mRNA species present in the initial population at a frequency of at least 1 in 200.

The protocol for RT-PCR-DD analysis has been also reported [8]. Briefly, total RNAs were extracted from intracellular bacteria during the infection of HeLa cells or control bacteria grown in cell culture medium, and were used as templates for first-strand cDNA synthesis in the presence of the primers DUS-IN, DUS-OUT, 26L-IN plus 27L-IN, 26L-OUT plus 27L-OUT, RS3-IN, RS3-OUT listed in Table 1. Then second-strand cDNAs synthesis was carried out using the corresponding oligonucleotides and a mixture of random hexamers as primers, and the PCR products were analyzed by polyacrylamide gel electrophoresis. The gel was stained by the silver staining method [44]. The slices of gel containing the differentially expressed transcripts were cut with a sharp scalpel, and the DNA fragments were allowed to diffuse into 100 μl of sterile water at 37°C for 4 h under stirring. The eluted DNA was ethanol precipitated using glycogen as a carrier. Then, it was used as a template in a PCR with the corresponding primers. The positive amplicons were cloned in a pGEM easy vector (Promega) and sequenced.

RT-PCR slot blot analysis of meningococcal *priA*-specific transcripts during infection of HeLa cells was performed as previously described [8]. To prepare gene-specific cDNA probes, the RNAs were subjected to reverse transcription using the oligonucleotides NMB0551-2 or 16Suniv-2 (loading control) as primers. The labeling procedure consisted of a PCR, where 2 μl of cDNA was used as template in a 50 μl mixtures containing 0.2 mM of each dCTP and dTTP, 2.5 μM of each dATP and dGTP, 0.12 μM [α-^32^P]ATP (3000 Ci mmol^-1^) and [α-^32^P]GTP (3000 Ci mmol^-1^), 0.2 μM forward NMB0551-1 or 16Suniv-1 primers, and 0.05 U μl^-1 ^of Taq polymerase (Perkin Elmer S.p.A). The amplification reaction consisted of 20–25 cycles including 45 sec of denaturation at 94°C, 45 sec of annealing at 55°C and 45 sec of extension at 72°C. The number of cycles was critical to operate in the linear range of the PCR, and it was determined in preliminary experiments. The ^32^P-labeled cDNA probes were hybridized to different amounts (0.05 to 100 ng) of denatured NMB0551 (*priA*)- or 16S rRNA gene-specific DNA fragments generated by PCR with the corresponding primer pairs NMB0551-1/NMB0551-2, and 16Suniv-1/16Suniv-2, and fixed onto positively charged Hybond-N+ nylon membranes. The hybridization reactions were carried out in Church buffer [44] at 63°C. Semi-quantitative analysis was performed by directly counting the radioactivity bands by a PhosphoImager SI (Molecular Dynamics, Inc., Sunnyvale, CA).

Semi-quantitative analysis of the *priA*-specific transcript, normalized to 16S rRNA, was also performed by real-time RT-PCR. Total RNAs (1 μg) from either intracellular or control bacteria grown in cell culture medium were reverse-transcribed by using random hexamer (2.5 μM) with Superscript RT (Invitrogen). About 0.1–1% of each RT reaction was used to run real-time PCR on a SmartCycler System (Cepheid) with SYBR^® ^Green JumpStart Taq ReadyMix (Sigma-Aldrich) and the primer pairs 16Suniv-1/16S-r (specific for 16S rRNA) and NMB0551-f/NMB0551-r (specific for *priA*). PCR products were 185 bp for 16Suniv-1/16S-r, and 143 bp for NMB0551-f/NMB0551-r. Real-time PCR samples were run in triplicate. The real-time PCR conditions were: 30 sec at 94°C, 30 sec at 55°C, 30 sec at 72°C for 35 cycles; detection of PCR products was performed at 83°C.

### Bacteria viability assay

The viability of meningococcal strains was determined by using the Live/Dead Bac*Light *bacterial viability kit (Molecular Probe). To this purpose, meningococci were grown to either middle (0.5 OD_600 nm_) or late (1.0 OD_600 nm_) logarithmic phase in 50 ml of GC medium with 1% Polyvitox as described above. Then 1 ml of bacterial suspension was collected by low-speed centrifugation. Meningococci were washed, re-suspended in 1 ml 0.9% NaCl and mixed with an equal volume of 2X working solution of Live/Dead Bac*Light *containing a 1:1 mixture of SYTO9 and propidium iodide. After 15 min dark incubation, 5 μl of mounted specimens were viewed with a Nikon Optiphot-2 microscope with an episcopic-fluorescence attachment (EFD-3, Nikon).

### Hydrogen peroxide and sodium nitroprusside sensitivity assays

B1940, B1940ΩpriA and B1940ΩpriA/priA+ were grown to middle logarithmic phase (0.5 OD_600 nm _corresponding to about 1 × 10^9 ^CFU). Then, 5 ml aliquots ("input" CFU = 5 × 10^9 ^CFU for each strains) were placed into 15-ml conical tubes. H_2_O_2 _was added to the tubes at final concentrations of 0, 0.5, or 1 mM, and tubes were incubated with shaking for 20 min at 37°C. Cultures were immediately centrifuged and pellets were re-suspended into 5 ml GC broth. These re-suspensions were serially diluted into GC broth and spotted onto GC agar. The plates were incubated at 37°C in a 5% CO_2 _incubator and the colonies were counted after 24 h of growth. For the sodium nitroprusside sensitivity assay, dilutions of freshly grown liquid cultures of *N. meningitidis *to middle logarithmic phase (0.5 OD_600 nm _corresponding to about 1 × 10^9 ^CFU) were immediately spread onto GC agar plates containing 1% Polyvitox and the nitric oxide generator. Plates were incubated in an atmosphere of 5% CO_2 _at 37°C.

### Immunufluorescence microscopy

Immunofluorescence analysis was performed as previously described to distinguish between extracellular and intracellular bacteria [7]. In brief, after infection, cells were washed once with PBS, and fixed with 3% paraformaldehyde. For staining of extracellular bacteria, incubation with primary antibodies was carried out for 20 min at room temperature. After washes in PBS, cells were incubated with secondary antibodies for 20 min in the dark, at room temperature. Subsequently, cells were permeabilized with saponin, incubated with primary antibodies to stain bacteria or intracellular markers and then incubated with secondary antibodies. Rabbit polyclonal anti-*N. meningitidis *antibody was from ViroStat while monoclonal H4A3 anti-LAMP1 was obtained from the Developmental Studies Hybridoma Bank at the University of Iowa. Primary and secondary antibodies were used at a 1:500 dilution. Mounted coverslips were examined using a Nikon Optiphot-2 microscope with an episcopic-fluorescence attachment (EFD-3, Nikon). Image processing was carried out with Adobe Photoshop version 7.0.

### Scanning electron microscopy

Meningococcal strains B1940 and B1940ΩpriA were grown to late (1.0 OD_600 nm_) logarithmic phase in 50 ml of GC medium with 1% Polyvitox as described above. Then 1 ml of bacterial suspension was collected by centrifugation. For SEM observations, meningococci were fixed with 1% glutaraldehyde, washed three times with distilled water by centrifugation, dehydrated in a graded alcohol series and critical-point dried. The sample was then mounted on Aluminum stubs, sputter-coated with gold and examined at an accelerating voltage of 20 kV with a Jeol 6060LV Scanning Electron microscope.

## Authors' contributions

AT and MDS carried out the experiments, CB participated in the design of the study and its coordination, PA conceived the study and wrote the manuscript. All authors read and approved the final manuscript.

## Competing interests

The authors declare that they have no competing interests.

## References

[B1] Merz AJ, So M (2000). Interactions of pathogenic *Neisseriae *with epithelial cell membranes. Annu Rev Cell Dev Biol.

[B2] Dehio C, Gray-Owen SD, Meyer TF (2000). Host cell invasion by pathogenic *Neisseriae*. Subcell Biochem.

[B3] Nassif X, Bourdoulous S, Eugène E, Couraud PO (2002). How do extracellular pathogens cross the blood-brain barrier?. Trends Microbiol.

[B4] Tinsley C, Nassif X (2001). Meningococcal pathogenesis: at the boundary between the pre- and post-genomic eras. Curr Opin Microbiol.

[B5] Tzeng YL, Stephens DS (2000). Epidemiology and pathogenesis of *Neisseria meningitidis*. Microbes Infect.

[B6] Hauck CR, Meyer TF (2003). 'Small' talk: Opa proteins as mediators of *Neisseria *– host-cell communication. Curr Opin Microbiol.

[B7] Spinosa MR, Progida C, Talà A, Cogli L, Alifano P, Bucci C (2007). The *Neisseria meningitidis *capsule is important for intracellular survival in human cells. Infect Immun.

[B8] Monaco C, Talà A, Spinosa MR, Progida C, De Nitto E, Gaballo A, Bruni CB, Bucci C, Alifano P (2006). Identification of a meningococcal L-glutamate ABC transporter operon essential for growth in low-sodium environments. Infect Immun.

[B9] Pagliarulo C, Salvatore P, De Vitis LR, Colicchio R, Monaco C, Tredici M, Talà A, Bardaro M, Lavitola A, Bruni CB, Alifano P (2004). Regulation and differential expression of *gdhA *encoding NADP-specific glutamate dehydrogenase in *Neisseria meningitidis *clinical isolates. Mol Microbiol.

[B10] Schekman R, Weiner JH, Weiner A, Kornberg A (1975). Ten proteins required for conversion of phiX174 single-stranded DNA to duplex form *in vitro*. Resolution and reconstitution. J Biol Chem.

[B11] Wickner S, Hurwitz J (1974). Conversion of phiX174 viral DNA to double-stranded form by purified *Escherichia coli *proteins. Proc Natl Acad Sci USA.

[B12] Marians KJ (1999). PriA: at the crossroads of DNA replication and recombination. Prog Nucleic Acid Res Mol Biol.

[B13] Lovett ST (2003). Connecting replication and recombination. Mol Cell.

[B14] Lovett ST (2005). Filling the gaps in replication restart pathways. Mol Cell.

[B15] de Jonge MI, Hamstra HJ, van Alphen L, Dankert J, Ley P van der (2003). Mapping the binding domains on meningococcal Opa proteins for CEACAM1 and CEA receptors. Mol Microbiol.

[B16] Massari P, King CA, Ho AY, Wetzler LM (2003). Neisserial PorB is translocated to the mitochondria of HeLa cells infected with *Neisseria meningitidis *and protects cells from apoptosis. Cell Microbiol.

[B17] Pridmore AC, Wyllie DH, Abdillahi F, Steeghs L, Ley P van der, Dower SK, Read RC (2001). A lipopolysaccharide-deficient mutant of *Neisseria meningitidis *elicits attenuated cytokine release by human macrophages and signals via toll-like receptor (TLR) 2 but not via TLR4/MD2. J Infect Dis.

[B18] Pridmore AC, Jarvis GA, John CM, Jack DL, Dower SK, Read RC (2003). Activation of toll-like receptor 2 (TLR2) and TLR4/MD2 by *Neisseria *is independent of capsule and lipooligosaccharide (LOS) sialylation but varies widely among LOS from different strains. Infect Immun.

[B19] Virji M, Evans D, Hadfield A, Grunert F, Teixeira AM, Watt SM (1999). Critical determinants of host receptor targeting by *Neisseria meningitidis *and *Neisseria gonorrhoeae*: identification of Opa adhesiotopes on the N-domain of CD66 molecules. Mol Microbiol.

[B20] Wyllie DH, Kiss-Toth E, Visintin A, Smith SC, Boussouf S, Segal DM, Duff GW, Dower SK (2000). Evidence for an accessory protein function for Toll-like receptor 1 in anti-bacterial responses. J Immunol.

[B21] Dietrich G, Kurz S, Hubner C, Aepinus C, Theiss S, Guckenberger M, Panzner U, Weber J, Frosch M (2003). Transcriptome analysis of *Neisseria meningitidis *during infection. J Bacteriol.

[B22] Tzeng YL, Datta AK, Strole CA, Lobritz MA, Carlson RW, Stephens DS (2005). Translocation and surface expression of lipidated serogroup B capsular Polysaccharide in *Neisseria meningitidis*. Infect Immun.

[B23] Schmitt C, Turner D, Boesl M, Abele M, Frosch M, Kurzai O (2007). A functional two-partner secretion system contributes to adhesion of *Neisseria meningitidis *to epithelial cells. J Bacteriol.

[B24] Lee EH, Kornberg A (1991). Replication deficiencies in *priA *mutants of *Escherichia coli *lacking the primosomal replication n' protein. Proc Natl Acad Sci USA.

[B25] Shaw JH, Falkow S (1988). Model for invasion of human tissue culture cells by *Neisseria gonorrhoeae*. Infect Immun.

[B26] Williams JM, Chen GC, Zhu L, Rest RF (1998). Using the yeast two-hybrid system to identify human epithelial cell proteins that bind gonococcal Opa proteins: intracellular gonococci bind pyruvate kinase via their Opa proteins and require host pyruvate for growth. Mol Microbiol.

[B27] Lin L, Ayala P, Larson J, Mulks M, Fukuda M, Carlsson SR, Enns C, So M (1997). The *Neisseria *type 2 IgA1 protease cleaves LAMP1 and promotes survival of bacteria within epithelial cells. Mol Microbiol.

[B28] Smith HO, Gwinn ML, Salzberg SL (1999). DNA uptake signal sequences in naturally transformable bacteria. Res Microbiol.

[B29] Stryer L (1988). Biochemistry.

[B30] Hill JC (1971). Effect of glutamate on exogenous citrate catabolism of *Neisseria meningitidis *and of other species of *Neisseria*. J Bacteriol.

[B31] Cox MM, Goodman MF, Kreuzer KN, Sherratt DJ, Sandler SJ, Marians KJ (2000). The importance of repairing stalled replication forks. Nature.

[B32] Kuzminov A (1999). Recombinational repair of DNA damage in *Escherichia coli *and bacteriophage lambda. Microbiol Mol Biol Rev.

[B33] Nurse P, Zavitz KH, Marians KJ (1991). Inactivation of the *Escherichia coli *priA DNA replication protein induces the SOS response. J Bacteriol.

[B34] Kline KA, Seifert HS (2005). Mutation of the *priA *gene of *Neisseria gonorrhoeae *affects DNA transformation and DNA repair. J Bacteriol.

[B35] Seib KL, Tseng HJ, McEwan AG, Apicella MA, Jennings MP (2004). Defenses against oxidative stress in *Neisseria gonorrhoeae *and *Neisseria meningitidis*: distinctive systems for different lifestyles. J Infect Dis.

[B36] Overton TW, Whitehead R, Li Y, Snyder LA, Saunders NJ, Smith H, Cole JA (2006). Coordinated regulation of the *Neisseria gonorrhoeae*-truncated denitrification pathway by the nitric oxide-sensitive repressor, NsrR, and nitrite-insensitive NarQ-NarP. J Biol Chem.

[B37] Rock JD, Mahnane MR, Anjum MF, Shaw JG, Read RC, Moir JW (2005). The pathogen *Neisseria meningitidis *requires oxygen, but supplements growth by denitrification. Nitrite, nitric oxide and oxygen control respiratory flux at genetic and metabolic levels. Mol Microbiol.

[B38] De Groote MA, Granger D, Xu Y, Campbell G, Prince R, Fang FC (1995). Genetic and redox determinants of nitric oxide cytotoxicity in a *Salmonella typhimurium *model. Proc Natl Acad Sci USA.

[B39] Schapiro JM, Libby SJ, Fang FC (2003). Inhibition of bacterial DNA replication by zinc mobilization during nitrosative stress. Proc Natl Acad Sci USA.

[B40] Stevanin TM, Moir JWB, Read RC (2005). Nitric oxide detoxification systems enhance survival of *Neisseria meningitidis *in human macrophages and in nasopharyngeal mucosa. Infect Immun.

[B41] Eskelinen EL (2006). Roles of LAMP-1 and LAMP-2 in lysosome biogenesis and autophagy. Mol Aspects Med.

[B42] Frosch M, Schultz E, Glenn-Calvo E, Meyer TF (1990). Generation of capsule-deficient *Neisseria meningitidis *strains by homologous recombination. Mol Microbiol.

[B43] Bucci C, Lavitola A, Salvatore P, Del Giudice L, Massardo DR, Bruni CB, Alifano P (1999). Hypermutation in pathogenic bacteria: frequent phase variation in meningococci is a phenotypic trait of a specialized mutator biotype. Mol Cell.

[B44] Sambrook J, Russell DW (2001). Molecular Cloning. A Laboratory Manual.

[B45] Salvatore P, Cantalupo G, Pagliarulo C, Tredici M, Lavitola A, Bucci C, Bruni CB, Alifano P (2000). A new vector for insertion of any DNA fragment into the chromosome of trasformable *Neisseria*. Plasmid.

